# Suicidal Presentations to Emergency Departments in a Large Australian Public Health Service over 10 Years

**DOI:** 10.3390/ijerph17165920

**Published:** 2020-08-14

**Authors:** Nicolas J.C. Stapelberg, Jerneja Sveticic, Ian Hughes, Kathryn Turner

**Affiliations:** 1 Mental Health and Specialist Services, Gold Coast Hospital and Health Services, Southport, QLD 4215, Australia; jerneja.sveticic@health.qld.gov.au (J.S.); Ian.Hughes2@health.qld.gov.au (I.H.); kathryn.turner@health.qld.gov.au (K.T.); 2 Faculty of Health Sciences & Medicine, Bond University, Robina, QLD 4226, Australia

**Keywords:** suicide, self-harm, emergency department, machine learning, Bayesian method

## Abstract

This paper presents trends and characteristics for 32,094 suicidal presentations to two Emergency Departments (EDs) in a large health service in Australia across a 10-year period (2009–2018). Prevalence of annual suicidal presentations and for selected groups of consumers (by sex, age groups, and ethnicity) was determined from a machine learning diagnostic algorithm developed for this purpose and a Bayesian estimation approach. A linear increase in the number of suicidal presentations over 10 years was observed, which was 2.8-times higher than the increase noted in all ED presentations and 6.1-times higher than the increase in the population size. Females had higher presentation rates than males, particularly among younger age groups. The highest rates of presentations were by persons aged 15–24. Overseas-born persons had around half the rates of suicidal presentations than Australian-born persons, and Indigenous persons had 2.9-times higher rates than non-Indigenous persons. Of all presenters, 70.6% presented once, but 5.7% had five or more presentations. Seasonal distribution of presentations showed a peak at the end of spring and a decline in winter months. These findings can inform the allocation of health resources and guide the development of suicide prevention strategies for people presenting to hospitals in suicidal crisis.

## 1. Introduction

Suicide is a global, complex phenomenon that results in an annual loss of more than 800,000 lives worldwide [1]. In Australia, 3046 people died by suicide in 2018, accounting for a standardized suicide rate of 12.1 per 100,000 [2]. Intentional self-harm was ranked as the 13th leading cause of death across all ages and represented 1.9% of total mortality in Australia. Approximately one third of deaths among people aged under 35 years was attributed to suicide [2].

The World Health Organization [1] has recommended regular monitoring of suicide rates as the backbone to effective national suicide prevention strategies. While many countries have national systems that record, collect and process information related to suicide (albeit with varying degrees of accuracy due to the challenges related to misclassifications of true causes of death [1]), very few have equivalent systems specifically dedicated to non-fatal suicidal behaviors [3]. The true prevalence of a range of suicidal behaviors in the general population therefore remains difficult to gauge and is further obfuscated by the fact that most people never seek help for suicide-related concerns [4]. A large community survey conducted in Australia reported a ratio of completed:attempted suicide to be 1 to 23 [5].

One of the most frequently utilized methods of estimating the incidence of non-fatal suicidal behavior is by noting such behavior on presentation to health facilities, most typically Emergency Departments (EDs), even if these account for only a subset of the actual community incidence [6,7,8]. Trends of presentations with non-fatal suicidal behavior to one Australian hospital (which represents a part of the health service investigated in the present paper) were recently analyzed by Kõlves et al. [9]. The authors found a significant increase in the age-standardized rates of non-fatal suicidal presentations for both males and females, and particularly among the youth. Further, 13.4% of the sample re-presented with within one year after their index episode [9]. Studies with longer follow-up periods reported higher rates of repeated non-fatal suicidal behaviors, such as 21.6% in a study of patients followed for 10 years [10]. Presentations with suicidal ideation have a higher rate of re-presentations than suicide attempts [11] and are more frequent among males than females [11,12]. Racial and ethnic differences have also been found to impact on rates of suicidal presentations to EDs, with evidence of members of ethnic minority groups displaying a higher risk of suicide yet being less likely to engage with mental health services [13,14]. 

Recent studies analyzed trends in hospital presentations combining suicidal ideation and attempts by identifying study populations through relevant diagnostic codes or presenting complaints assigned to ED encounters [15,16,17]. However, there is evidence that ED administrative data under-estimate the true volume of suicidal presentations by around 60% due to heterogeneity of their coding [18,19], prompting calls to develop more sensitive processes to improve their identification [20]. 

In 2016, the Gold Coast Hospital and Health Service (GCHHS) initiated a Suicide Prevention Strategy [21] based on the Zero Suicide Framework (ZSF) [22]. ZSF is a systems approach to suicide prevention underpinned by data-driven evaluation and continuous quality improvement, which is reliant on accurate and timely data acquisition. To overcome the above noted limitations inherent to the use of ED administrative data and to reduce high costs associated with manual identification of relevant cases, a machine learning algorithm was developed to identify suicidal presentations using presentation descriptions in the Emergency Department Information System (EDIS) database [23]. This algorithm was used to obtain data on suicidal presentations between 2009 and 2018 which were analyzed in this paper. The paper has four main aims:

- To analyze trends in suicidal presentations over a 10-year period;
- To analyze sociodemographic information of consumers with suicidal presentations (age, sex, ethnicity);
- To analyze the frequency of suicidal re-presentations; and
- To identify seasonal patterns of suicidal presentations.

## 2. Materials and Methods 

### 2.1. Context

This work was conducted within the GCHHS which provides public mental health services for a population of around 600,000 people [24]. GCHHS has two EDs (Gold Coast University Hospital and Robina Hospital) that represent the largest primary points of presentation for persons of all ages at risk of suicide. A cohort of all suicidal presentations to EDs between 1 January 2009 and 31 December 2018 was examined (*N* = 1,402,518).

### 2.2. Identification of Suicidal Presentations

The initial identification of suicidal cases was performed using a machine learning algorithm, the “Searching EDIS for Records of Suicidal Presentations” (SERoSP) [23]. SERoSP uses an evolutionary algorithm to weight 136 variables from a training dataset of EDIS data (March–May 2015), where suicidal presentations were identified by a psychiatrist. These variables included a range of diagnostic codes and presenting problems assigned at the point of ED triage such as Indigenous/non-Indigenous status of the consumer, score according to the Australian Triage Scale (5-point clinical tool used to establish the maximum waiting time for medical assessment and treatment of a patient), discharge destinations, and 52 different keywords indicative of the presentations being suicide-related. The program then “learned” from trial and error, over 150 successive generations, using 100 variations of variable weights per generation, to detect suicidal presentations with a sensitivity of 95% and a specificity of 92%. This algorithm was designated as SERoSP1. Further work was then undertaken to increase the sensitivity and specificity of the algorithm by emphasizing analysis of triage text using word and phrase search strategies, and employing basic Natural Language Processing techniques to identify key words and word combinations used in the triage text of suicidal presentations. The modified algorithm was retrained on 23,786 psychiatrist-assessed ED presentations (July–December 2015) and validated on 26,130 psychiatrist-assessed ED presentations (July–December 2017), achieving a sensitivity of 87% for a pre-set specificity of 99%. This algorithm is referred to as SERoSP2. 

Following a well-used concept in clinical medicine where a highly sensitive test is run as a screening test followed by a high-specificity test to then confirm or reject a diagnosis [25,26], SERoSP1 was run first to ’screen‘ cases, theoretically missing only few cases, but incorporating a substantial number of false positives, followed by the SERoSP2 which eliminated as many false positives as possible. The combination was tested against the 26,130 human-assessed ED validation dataset (July–December 2017) which the SERoSP1-SERoSP2 combination had not been exposed to before. This resulted in a specificity of 0.972 (95% confidence intervals (CI): 0.969–0.974) when the sensitivity was set at 0.968 (0.960–0.974). 

SERoSP algorithm was unable to discriminate between suicide attempts, non-suicidal self-injury, and suicidal ideation based on EDIS data. This is in part because EDIS data is entered before a detailed mental health assessment occurs and the information contained therein is less detailed than those recorded into consumers’ clinical notes upon a more thorough assessment by a mental health practitioner. Therefore, throughout the paper, the term “suicidal presentations” is used to describe all suicide and self-harm related incidents. 

### 2.3. Statistical Analysis 

Diagnostic tests are commonly used for prevalence surveys, however, unless the test is perfect (i.e., with sensitivity and specificity of 1), the observed prevalence must be adjusted to estimate the true prevalence [27,28]. Both traditional (frequentist) and Bayesian methods have been developed to estimate true prevalence. When the expected prevalence is low, assumptions required for frequentist approaches may not be met and unrealistic point estimates and wide confidence intervals result [27,29]. In this work, we use a Bayesian approach described by Joseph et al. [30] and utilized through Epitools (Bayesian estimation of true prevalence from survey testing with one test) [31]. The accuracy of Bayesian estimates is dependent on the quality of prior knowledge regarding sensitivity and specificity of the test and expectation of the prevalence [29]. Prior estimates of the sensitivity and specificity, in all cases, were those obtained from the SERoSP1-SERoSP2 diagnostic combination on the validation dataset. The prior estimate of the prevalence, in each case, was based on a crude adjustment of the observed prevalence based on the proportion of false positives seen in the validation dataset. Probability distributions of these prior estimates were based on the beta distribution with parameters, α and β, calculated from the point estimate and its 5%CI for sensitivity and specificity or, for prevalence, from the adjusted number of positives, x, and the population total, n (α = x + 1, β = n – x + 1) [30,31].

The posterior estimates of prevalence obtained are medians with 95% equally tailed credible intervals. The 95% credible interval (CDI) is the Bayesian equivalent to a 95% CI and is the interval in which the parameter (median in this instance) falls with 95% probability. The posterior probability densities, and hence CDIs, may be highly skewed [30]; hence, when the prevalence estimates are used to estimate the number of presentations, the sum of estimated presentations from subgroups may not necessarily equal the estimate calculated for the total. In this paper, medians and 95% CDIs are presented for annual estimates but only medians are shown for smaller subgroups.

Annual sex-based and age-specific rates were calculated using the Gold Coast estimated resident population figures for the years 2009–2018 [24]. For Indigenous population and country of birth figures, census data from 2011 and 2016 were used [24]. Comparisons between rates were performed using incident rate ratios (IRRs). Trend lines were examined using linear regression models, and annual percentage changes (APC) with 95%CI are presented. Presentation-based data is used in all analyses apart from the frequency of re-presentations, where person-based data is employed. The likelihood of re-presentations is compared between groups using odds ratios (OR). All analyses were performed using SPSS, Version 24 (IBM Corp., Armonk, NY, USA) [32]. 

### 2.4. Research Ethics

This work was performed as part of the project Gold Coast Mental Health and Specialist Services Suicide Prevention Strategy: Evaluation. It was recognized as Quality Activity by the GCHSS Human Research Ethics Committee and granted a research ethics exemption (LNR/2018/ QGC/47473).

## 3. Results

### 3.1. Rates of Suicidal Presentations

Between 2009 and 2018, 32,094 suicidal presentations to Gold Coast Emergency Departments were identified. Table 1 shows the numbers of annual suicidal presentations, alongside the 95% CDIs for the prevalence using the Bayesian estimates. On average, suicidal presentations accounted for 2.2% of all ED presentations, with a substantial increase in this proportion from 1.3% in 2009 to 3.1% in 2018.

The annual percentage change in the number of suicidal presentations was 13.5% (95%CI 11.2–15.8). This increase was 2.8-times higher than the increase noted in all ED presentations (APC = 4.8%, 95%CI 2.9–6.6), and 6.1-times higher than the increase in the population size (APC = 2.2%, 95%CI 2.0–2.4) during the same time. 

Combining presentations across the 10-year period, females had higher rates of suicidal presentations than males (594.8 vs. 542.5 per 100,000; IRR = 1.10, 95%CI 1.07–1.12). However, the increase in rates during the observed decade was larger for males (3.4-fold) than females (2.9-fold increase). For all persons, rates of suicidal presentations increased 3.0-fold between 2009 and 2018.

### 3.2. Demographic Characteristics 

**Figure 1:**
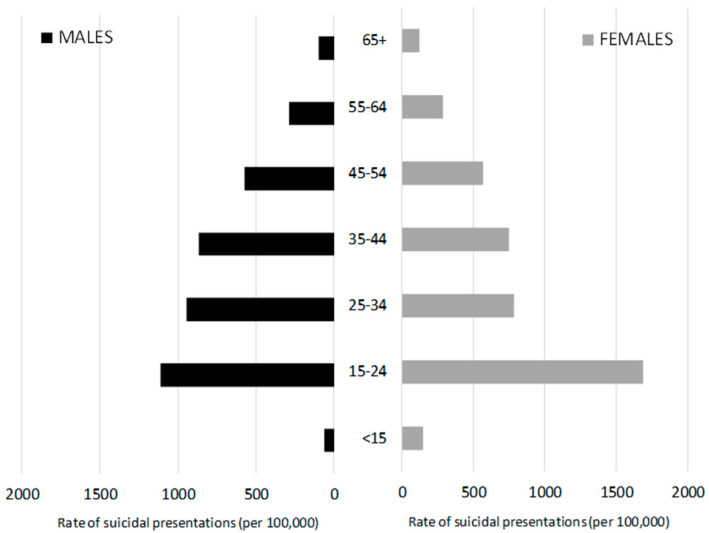
Age-specific rates of suicidal presentations by males and females, 2009–2018.

Figure 1 shows that the highest rates of presentations were by persons aged 15–24, which accounted for 37.4% of presentations by females and 28.1% of presentations by males. This was followed by those aged 25–34 (accounting for 25.2% of females’ presentations and 18.7% of males’) and 35–44 (22.3% of males’ and 17.7% of females’ presentations). Low rates of presentations are seen by older persons, with only 2.6% of all presentations by males and 3.2% of presentations by females in the 65+ age group. 

**Figure 2:**
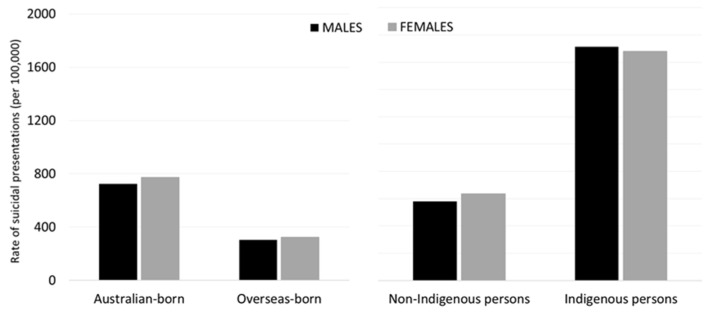
Rates of suicidal presentations by ethnicity groups, 2009–2018.

Across the 10-year period, 6646 suicidal presentations (20.7%) were by overseas-born persons, and 1363 (4.2%) were by persons identified as being of Indigenous background. Figure 2 compares rates of suicidal presentations for different ethnicity groups. Overseas-born persons had around half the rate of suicidal presentations as Australian-born persons (IRR = 0.44, 95%CI 0.42–0.45), with this ratio the same in males and females. Persons of Indigenous background had 2.8-times (95%CI 2.62–2.93) higher rates of suicidal presentations than non-Indigenous persons, with this difference more pronounced among males (IRR = 2.96, 95%CI 2.73–3.20) than females (IRR = 2.64, 95%CI 2.45–2.86).

### 3.3. Repeated Suicidal Presentations

As seen in Table 2, the total of 32,094 presentations over the 10-year period were attributed to 20,526 persons. The majority of persons (70.6%) presented once, 23.7% had 2–4 presentations, and 5.7% were considered to be “frequent presenters” [33] by having a total of five or more presentations during the observed 10-year period.

The frequency of repeated presentations differed between persons depending on their age of first presentation, country of birth, and Indigenous status, but not sex. A greater likelihood to have more than one suicidal presentation was noted in persons aged less than 15 years (OR = 1.44, 95%CI 1.26–1.66) and those aged 15–24 years (OR = 1.27, 95%CI 1.19–1.35), when compared to other age groups. In contrast, persons whose first presentation was when aged 65 or more were more likely to only have only one presentation during the observed 10 years (OR = 2.93, 95%CI 2.42–3.55). Australian-born persons were 1.42-times more likely to have multiple presentations than overseas-born persons (95%CI 1.32–1.53), and Indigenous persons were 1.61-times (95%CI 1.38–1.87) more likely to have multiple presentations than non-Indigenous persons. The two groups of persons with the highest likelihood of being frequent presenters were youth under 15 years (11.1%) and Indigenous Australians (9.8%).

### 3.4. Seasonality of Suicidal Presentations

**Figure 3:**
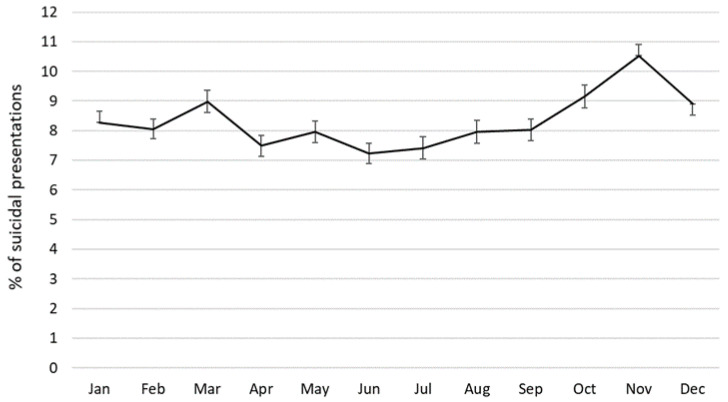
Seasonal distribution of suicidal presentations, 2009–2018.

Figure 3 shows the distribution of suicidal presentations across months, combining data for all 10 years. The highest percentage of presentations occurred between October and December, with a distinct peak in November (10.5%, 95%CI 10.1–10.9), which marks the end of spring in the southern hemisphere. This was followed by another, though smaller increase in March (start of autumn) with 9.0% (95%CI 8.6–9.4) of all suicidal presentations. The lowest percentage of suicidal presentations occurred in winter months (June and July) which accounted for 7.2% (95%CI 6.9–7.6) and 7.4% (95%CI 7.0–7.8) of suicidal presentations, respectively. No differences in the monthly distribution of suicidal presentations were observed between males and females (χ2(11) = 12.25, *p* = 0.345).

## 4. Discussion

The use of a machine learning algorithm for the identification of suicidal presentations in ED administrative data introduces an innovative methodological approach to epidemiological suicidology. Results showed that rates of suicidal presentations to EDs in a large Australian public health service have tripled over a 10-year period; this increase was significantly greater than the overall increase in population size and in total ED presentations over the same period. This finding replicates similar results observed in New South Wales, Australia [17], and adds to the well-established literature on such increases when considering only suicide attempts [9,34]. 

Whilst it is possible that the observed trends reflect realistic increases in the number of persons experiencing suicidal crises in the geographical area included in the present analysis, reasons for it (such as potential sequalae of the 2007 economic recession as observed by Milner et al. [35]) could not be explored here. Other contributing factors need to be considered, such as attitudinal changes related to help-seeking behaviors, changes in referral pathways and models of care, or the (un)availability of alternative sources of support for acutely suicidal persons [36,37,38]. Finally, it is possible that the introduction of training programs, such as Suicide Risk Assessment and Management in Emergency Department Settings (SRAM-ED) program in 2015 [39], have improved the recording of relevant clinical details pertaining to suicidality in ED triage notes, increasing their probability of identification by the machine learning algorithm used in this work.

Demographic and socioeconomic variables associated with suicide risk have been the subject of many epidemiological studies, though typically limited to deaths by suicide or suicide attempts [40,41]. In this work, which combined presentations with suicidal ideation and suicide attempts, rates by females were found to be higher than by males. This is in contrast with findings by Perera, Wand, Bein, Chalkley, Ivers, Steinbeck, Shields, and Dinh [17] who noted a nearly equal division between males and females. A previously published analysis of a subset of presentations included in this paper [18] found that in 2017, 63.4% of all suicidal presentations to ED were due to suicidal ideation. Considering the emerging evidence that males have higher rates of ED presentations with suicidal ideation [11], it could be expected that males would account for a larger proportion of suicidal presentations compared to females in our sample, however, the inability to differentiate between types of presentations prevented a more in-depth exploration of these trends. 

Persons aged 15–24 accounted for the largest number and had the highest rate of suicidal presentation across the 10 years. Recent increases in suicide attempts by young people, and in particularly young men, have previously been observed [9]. Some authors have linked this to earlier age of onset of puberty and with it, earlier onset of depression and other mental health concerns, increased exposure to media portrayals of suicide, and increased use of social media which may facilitate the development of suicidal thoughts and attempts [42,43]. As noted above, a differential analysis of trends of suicide ideation and suicide attempts in different age groups is required to allow for identification of vulnerable groups that require most urgent responding. 

Overseas-born persons’ rate of suicidal presentation was about half of that observed for Australian-born people. This follows the previously observed disparity between suicide rates of first-generation migrants in Queensland, which are significantly lower than those of Australian-born persons [44]. Differences between the two groups was higher than reported in the analysis of deaths by suicide by Kolves and De Leo [44], which could be attributed to immigrants’ reluctance to utilize mental-health services for suicide-related concerns [45]. Similarly, many Indigenous people experience significant barriers in accessing culturally appropriate support for mental health more broadly, and in particular for suicidality [46], which is believed to contribute to the 2.2-times higher suicide rate in Indigenous persons compared to non-Indigenous persons [47]. Despite the previously argued reluctance by many Indigenous Australians to engage with Western-based medical models of treatment [48], their rates of suicidal presentations to our EDs remained much higher than those of non-Indigenous persons. It is possible that among presentations by Indigenous persons included in our analysis, a greater proportion resulted from suicide attempts, particularly if using methods that are more likely to require medical interventions [14] or were involuntary in nature [49]. 

High repetition rates of suicidal presentations have previously been noted in Australian [9] and Irish [3] studies of hospital presentations with suicide attempts, although differences in follow-up times and the inclusion of suicidal ideation in our analyses prevent direct comparisons of findings. We contribute to the existing literature a delineation of the risk of re-presentation for different demographic and ethnic groups. Persons that were most likely to have repeated presentations belong to the same groups that had the highest rates of suicidal presentations: youth and Indigenous Australians. To a degree, this may be a consequence of different units of measurements (presentation-level when calculating rates, and person-level when analyzing re-presentations), and the fact that some older people presenting with suicidality may have died before the completion of the 10 year follow up period. 

Analysis of monthly distribution of suicidal presentations to EDs identified a distinct peak at the end of spring (in November) and the lowest frequency during winter months. This finding partially aligns with previously observed seasonal patterns in deaths by suicide in Australia, which typically peak in spring/early summer and dip in autumn (for females) and winter (for males) [50,51]. However, some studies have found the distribution of non-fatal suicidal behaviors across the year to be less pronounced, arguably due to the predominant use of low-risk methods that have the least pronounced seasonal variations [41], such as cutting and poisoning, in suicide attempts. Potential variations in the prevalence of suicidal ideation, specifically within the subset of the population that access ED for help, currently remains a little researched topic outside of the debate on seasonal depression, which is believed to be less prominent in tropic areas of the southern hemisphere [52]. It is possible that other factors contributed to the observed trends in our study, such as the large numbers of domestic and international travelers visiting the Gold Coast at different times of the year, which has previously been found to have a significant impact on the volume of ED presentations [53]. The observed peak in suicidal presentations in November could therefore be partly due to the mass gathering of high school leavers (“schoolies”) in the area, which has been linked to an increase in the number of mental health related presentations to ED during that time [54]. A more detailed investigation of seasonal trends of different types of suicidal presentations, further separated by sex and age groups, is required to disentangle the effects of these associations. 

### Limitations 

There are limitations of this work that warrant consideration. Any diagnostic test used for surveillance purposes that is not perfect will provide prevalence estimates that come with error. The combined SERoSP1-SERoSP2 test had high levels of sensitivity and specificity, however, when applied to rare events, even tests as accurate as this produce large numbers of false positives and hence require an adjustment of the observed prevalence. The Bayesian method used in this work provided the best available estimates but is very dependent on high quality prior information. For the purposes of this analysis, it was assumed that the accuracy observed in the validation dataset (using 2017 data) provided a good estimate of the test’s performance in populations from other years and in specific subgroups. Similarly, it was assumed that the proportion of false positives seen in the validation dataset would be similar in other population groups, enabling a reasonably good prior estimate of prevalence. 

The size of the population for which the prevalence is being estimated also contributes to the accuracy of the estimate. This means it was not possible to accurately estimate the prevalence for some small populations (e.g., separate annual presentations by sex and age groups) nor could we obtain age-standardized rates that would allow for more meaningful comparisons between populations that are known to have different age-structured populations (most notably, Indigenous and non-Indigenous Australians). We acknowledge this as a limitation of this paper. It is also possible that the numbers of suicidal presentations by Indigenous people and first-generation migrants are under-estimated in our analysis, as has been noted in some earlier studies [55,56], particularly in the early years before GCHHS invested concentrated efforts towards improving the recording of sociodemographic characteristics of its consumers [57]. In analyzing re-presentations to ED, different periods of follow-up were applied to consumers depending on the year of their index presentation. Finally, we could not control for the possibility that some persons died during the follow-up period (particularly those of older age at the time of their index presentation) or have had repeated suicidal presentations to health services outside of the Gold Coast geographical catchment boundaries. 

The inability of the developed algorithm to differentiate between types of suicidal presentations presents a limitation of the current work. We note that while the method presented here is suitable for surveillance and as a means to efficiently track suicidal presentations to EDs over time, the estimates of prevalence come with varying degrees of error. Therefore, for more detailed and accurate case-level investigations, a manual investigation is still required.

## 5. Conclusions

Data on hospital attendances for all suicide-related concerns (rather than just suicide attempts) provides for a more complete picture of the overall burden suicidality places on public hospital systems. Importantly, such data can inform the development of suicide prevention strategies targeted to those with the highest rates of (re)presentations, such as youth and Indigenous persons. 

Findings of the present work offer additional pragmatic opportunities by enabling monitoring of the trends of suicide-related presentations, modelling of future demands, and informing a meaningful distribution of scarce health resources. The importance of timely access to data is particularly evident in times of anticipated changes in suicide risk experienced by broader communities, as seen with the spread of COVID-19 [58]. The fact that over the last 10 years, the increase in suicidal presentations has by far exceeded the increase seen for all ED presentations also highlights the need for consideration of alternative models of care to avoid the overwhelming of health systems [59]. 

Witt and Robinson [60] note the role machine-learning approaches can play in morbidity surveillance systems by improving timeliness and efficiency while approaching the quality of manualized case ascertainment. The use of the SERoSP algorithm, though not without its limitations, has demonstrated good utility in analyzing a large cohort of suicide-related ED presentations. 

## Figures and Tables

**Table 1:** Suicidal presentations to Gold Coast Emergency Departments, 2009–2018.

Year	Suicidal Presentations *N* (95% CDI)	All ED Presentations	Suicidal Presentations as % of All ED	Gold Coast Population	Rates of Suicidal Presentations (Per 100,000 Population)		
					Males	Females	Persons
2009	1446 (1334–1557)	111,201	1.3	495,835	258.0	299.8	291.6
2010	1652 (1542–1762)	110,114	1.5	506,135	294.0	334.6	326.3
2011	2089 (2090–2205)	116,055	1.8	515,202	374.9	411.3	405.5
2012	2467 (2468–2590)	123,346	2.0	528,342	406.9	501.0	466.9
2013	2798 (2664–2931)	133,231	2.1	540,687	450.6	533.7	517.5
2014	3376 (3230–3523)	146,800	2.3	550,718	568.1	630.5	613.1
2015	3756 (3599–3912)	156,487	2.4	561,629	597.6	709.6	668.7
2016	4246 (4083–4410)	163,319	2.6	575,303	698.0	720.7	738.1
2017	4884 (4716–5052)	168,416	2.9	591,356	785.7	809.0	825.9
2018	5380 (5207–5554)	173,549	3.1	606,774	873.4	871.4	886.7
2009–2018	32,094 (30,855–35,063)	1,402,521	2.2	5,471,981	542.5	594.8	586.5

Notes: CDI – Credible Interval. ED – Emergency Department.

**Table 2:** Frequency of suicidal presentations per person, 2009–2018.

	*N*^1^ (Persons)	1 Presentation *N*(%)	2–4 Presentations *N*(%)	5 or More Presentations *N*(%)
All persons	21,220	14,974 (70.6%)	5028 (23.7%)	1218 (5.7%)
Sex				
Males	10,341	7360 (71.2%)	2402 (23.2%)	579 (5.6%)
Females	11,249	7928 (70.5%)	2676 (23.8%)	644 (5.7%)
Age groups ^2^				
Under 15	886	558 (62.9%)	231 (26.0%)	98 (11.0%)
15–24	6752	4542 (67.3%)	1692 (25.1%)	519 (7.7%)
25–34	4738	3396 (71.7%)	1070 (22.6%)	273 (5.8%)
35–44	4000	2831 (70.8%)	978 (24.4%)	192 (4.8%)
45–54	2817	2030 (72.0%)	623 (22.1%)	165 (5.8%)
55–64	1291	967 (74.9%)	275 (21.3%)	49 (3.8%)
65 +	953	831 (87.2%)	112 (11.8%)	10 (1.1%)
Country of birth				
Australian-born	16,752	11,599 (69.2%)	4126 (24.6%)	1027 (6.1%)
Overseas-born	4789	3651 (76.2%)	943 (19.7%)	194 (4.1%)
Indigenous status				
Indigenous	746	450 (60.4%)	223 (29.8%)	73 (9.8%)
Non-Indigenous	20,568	14,605 (71.0%)	4817 (23.4%)	1146 (5.6%)

Notes: ^1^ – N refers to the sum of estimates of presentations for subgroups rather than the estimate for the whole group (see Methods for more details). This allowed for meaningful % values to be shown. ^2^ - When a person had more than one suicidal presentation, the age at their first presentation was included in the analysis.
